# Editorial: Case reports in paediatric orthopaedics 2022

**DOI:** 10.3389/fped.2023.1211236

**Published:** 2023-06-05

**Authors:** Martina Marsiolo, Angelo Gabriele Aulisa

**Affiliations:** ^1^ U.O.C Traumatology, Bambino Gesù Children's Hospital, IRCCS, Rome, Italy; ^2^ Department of Human, Social and Health Sciences, University of Cassino, Cassino, Italy

**Keywords:** case report, children, scoliosis, fibular graft, bone wax

**Editorial on the Research Topic** Case reports in pediatric orthopedics 2022

Paediatric orthopaedics is a very fascinating branch of orthopaedics as a child's skeleton has great remodelling capabilities thanks to growth cartilage, but often this can be the cause of growth defects when damaged. Sometimes, the pathologies have a clinical manifestation that involves different systems, and often in these cases, they are rare pathologies with difficult diagnosis. The diagnosis in children is made more difficult by the complicated communication with the patients and treatment of the same pathology varies according to the patient's age. These situations are already present for well-known pathologies and become even more serious in the case of rare pathologies or for atypical manifestations where lack of diagnosis and treatment can lead to poor prognosis. The issues covered by these studies are different, all united by the fact of being unusual and rare. This topic aims to share a series of rare case reports to form an understanding of the possible expression of various conditions and their treatments for improving clinical practice, diagnostics, and therapeutics.

Physeal fractures account for 30% of all paediatric fractures, although growth plate injuries are common. Growth disturbance related to physis lesions is rare, but when it occurs, it could cause severe deformities with poor prognosis; distal physis is more frequently involved than proximal physis, and distal tibial physis is the most commonly involved type of low extremity followed by the distal femur. The literature has reported that a maximum of 30% of the bone bridge of the physis area can be successfully removed, and in cases of genu valgus of 20°, a corrective osteotomy is often necessary (1). Basiglini et al. showed that partial epiphysiodesis inserting the bone wax is possible in up to 30% of the cartilage area without osteotomy, because the cartilage around the lesion can react by hyperactivating and allow the recovery of the load axis. Ankle fractures account for about 5% of all paediatric fractures. While rare, loss of the medial malleolus is more common in children. Furthermore, it leads to a higher risk of complications such as medial physeal bar with varus angulation and leg length discrepancies (LLD) due to premature physis arrest. Medial malleolus contributes to maintaining normal tibiotalar joint characteristics, and fractures can significantly decrease contact area, increase contact pressure, and lead to ankle instability (2). Chengming Zhu et al. reported that in rare cases of open Salter–Harris type VI fractures with complete loss of the medial malleolus, there is the possibility of rebuilding it using an autologous iliac crest apophyseal graft without deformity and LLD.

Primary osseous spinal tumours are rare in children and young adults, accounting for only 1% of all spine and spinal cord tumours combined (3), but they should always be considered in the differential diagnosis of back pain in children. Despite benign bone lesions prevailing in young patients (4), some cases of malignant lesions have been reported in the literature. In general, orthopaedic surgeons use age and anatomical site to determine the histological type of tumour, but it is important not to use these as fixed rules because sometimes, in orthopaedic oncology, these are subverted, and often when it happens, they have more aggressive behaviour; the lack of diagnosis leads to a poor prognosis. Rosa M. Egea-Gámez et al. described the first published case of giant cell-rich osteosarcoma (GCRO) in the cervical spine in a paediatric patient, reporting a treatment option that showed excellent results at 3 years of follow-up; in this case report, the age, site of the lesion, and histological variant were unique.

Florio et al. reported a successful reconstruction of the metatarsal bone with fibular graft for a case of giant cell tumour (GCT) marked by an unusual location and age group. GCT develops almost exclusively in the epiphysis of long bones, and the most common location is around the knee region; only four cases of GCT on metatarsal bone have been reported in the literature (5).

Heterotopic ossification (HO) is a rare condition that is generally secondary to other conditions, particularly trauma (often repetitive microtrauma) or hereditary form; it is essential to make a differential diagnosis with oncological pathologies to better manage these conditions (6). This pathology can rarely occur without a previous condition in an atypical location. It is important to know this, especially because early HO lesions may histologically mimic a sarcoma; unlike the latter, in the case of HO, complete removal allows excellent results without recurrences. Instead, when it is secondary to a genetic condition such as fibrodysplasia ossificans progressive (FOP), the prognosis assumes an aggressive and relapsing aspect especially due to surgical treatment; it is essential in these cases to intervene surgically only when necessary. Dong Sun at al. reported a case report of FOP where the surgical choice was adequately made in respect to the affected joints essential for leading a satisfactory life (jaw, hip, and spine). This is the first case of non-traumatic massive heterotopic iliopsoas ossification described in the literature.

Kaposi haemangioendothelioma (KHE) is a rare vascular neoplasm that presents usually within the first year of life. There is often a delay in diagnosis, and this may lead to morbidity and mortality of up to 30%, mostly due to a life-threatening consumptive coagulopathy named the Kasabach–Merritt phenomenon (KMP), which occurs in 40%–70% of cases, although this is a complication. However, when present, it helps in the diagnosis (7). The treatment is still much discussed, especially from a pharmacological point of view. Qiu et al. reported a case report of KHE at a late age originating from the bone and limited inside the bone with bilateral involvement. It is important to note that when involved, only the bone without periosteal reaction KMP is not associated, and this can lead to a lack of diagnosis. This is the only case involving bilateral femur symmetry reported in the literature. In this case, sirolimus without prednisolone was effective. Given the rarity of the pathology, it may happen that despite the presence of KMP, there may be a delay in the diagnosis. In this topic, a case report of KHE with KMP in a 2-month patient has been reported; he showed symptoms from the first week of life and was treated first for urticaria, then cellulitis, and then necrotic fasciitis until the right diagnosis was made, at two months of age. He was treated with sirolimus (off-therapy because it is accepted from 2 years onwards) with excellent results 3 years after treatment.

Congenital bowing of the tibia is a rare condition (1/140.000–190.000) and is considered the precursor of congenital pseudarthrosis of the tibia; little is known about its clinical features. While the diagnosis is simple, the treatment, which varies from conservative to surgical, is not. Surgical treatment could consist of different methods, none of which have shown superiority over the other, with a 50% amputation incidence. Mastantuoni et al. reported a case of congenital bowing of the tibia treated with double osteotomy and Tens, with excellent results after 8 years of treatment. Yijun Zhou et al. conducted the only epidemiological study, which included a large sample of patients (514). The authors reported a higher incidence of Crawford IV in boys and in the middle or distal part of the tibia; most patients were less than 3 years old, and the major surgical complications were ankle valgus and limb length discrepancy.

Osteogenesis imperfecta is an autosomal dominant congenital pathology characterised by bone fragility and associated with other clinical signs. Many gene variants determine a different phenotypic expression. In their study, Dirani et al. reported the first clinical description of a patient with a splice variant in intron 34 in the COL1A1.

Scoliosis is the most common congenital pathology, which can be caused by vertebra segmentation or formation defects. Caredda et al. showed how conservative treatment in the case of lumbar hemivertebra is a good choice; it can improve the Cobb's value of the curve and maintain the long-term correction. Conservative treatment in selected patients can also change the natural history of congenital scoliosis due to the failure of formation, which permits the hypertrophy of adjacent vertebrae, thus preventing its deformation. However, the treatment should be implemented as early as possible without waiting for the evolution of the curve, in contrary to what occurs in lower idiopathic scoliosis.

Scapholunate dissociation (SLD) is a very rare condition, especially in children. When it occurs, in most cases it is post-traumatic or secondary to other conditions. If undiagnosed, it can lead to chronic pain, SL advanced collapse, and early osteoarthritis (8). In this topic, Bandinelli et al. reported the only case known in the literature of SLD in a paediatric patient that was not secondary to trauma or other conditions. The unique symptom was a wrist extension deficit. The only alteration found in the patient was generalised hyperlaxity. Thanks to the correct diagnosis and treatment, the patient showed a complete functional recovery after 12 months. It is important to know that a trauma-free SLD can exist, probably due to hyperlaxity, which allows to treat it adequately while avoiding long-term complications.
